# Diverse diazotrophs are present on sinking particles in the North Pacific Subtropical Gyre

**DOI:** 10.1038/s41396-018-0259-x

**Published:** 2018-08-16

**Authors:** Hanna Farnelid, Kendra Turk-Kubo, Helle Ploug, Justin E. Ossolinski, James R. Collins, Benjamin A. S. Van Mooy, Jonathan P. Zehr

**Affiliations:** 1 Ocean Sciences Department, University of California at Santa Cruz, Santa Cruz, CA USA; 2 Centre for Ecology and Evolution in Microbial Model Systems, Linnaeus University, Kalmar, Sweden; 3 Department of Marine Sciences, University of Gothenburg, Gothenburg, Sweden; 4 Department of Marine Chemistry & Geochemistry, Woods Hole Oceanographic Institution, Woods Hole, MA USA; 5 MIT/WHOI Joint Program in Oceanography, University of Washington, Seattle, WA USA; 6 School of Oceanography and eScience Institute, University of Washington, Seattle, WA USA

**Keywords:** Microbial ecology, Microbial ecology, Ecosystem ecology

## Abstract

Sinking particles transport carbon and nutrients from the surface ocean into the deep sea and are considered hot spots for bacterial diversity and activity. In the oligotrophic oceans, nitrogen (N_2_)-fixing organisms (diazotrophs) are an important source of new N but the extent to which these organisms are present and exported on sinking particles is not well known. Sinking particles were collected every 6 h over a 2-day period using net traps deployed at 150 m in the North Pacific Subtropical Gyre. The bacterial community and composition of diazotrophs associated with individual and bulk sinking particles was assessed using 16S rRNA and *nifH* gene amplicon sequencing. The bacterial community composition in bulk particles remained remarkably consistent throughout time and space while large variations of individually picked particles were observed. This difference suggests that unique biogeochemical conditions within individual particles may offer distinct ecological niches for specialized bacterial taxa. Compared to surrounding seawater, particle samples were enriched in different size classes of globally significant N_2_-fixing cyanobacteria including *Trichodesmium*, symbionts of diatoms, and the unicellular cyanobacteria *Crocosphaera* and UCYN-A. The particles also contained *nifH* gene sequences of diverse non-cyanobacterial diazotrophs suggesting that particles could be loci for N_2_ fixation by heterotrophic bacteria. The results demonstrate that diverse diazotrophs were present on particles and that new N may thereby be directly exported from surface waters on sinking particles.

## Introduction

Marine environments exhibit microscale gradients of organic carbon and nutrients, representing diverse microniches for microorganisms to exploit. Particulate organic matter (POM) is ubiquitous in seawater and composed of a mixture of living microorganisms and dead detrital matter, providing ‘hotspots’ for microbial activity and organic matter remineralization that significantly contribute to biogeochemical element cycling rates [1]. Sinking particles transport organic and inorganic material into the deep ocean, a process known as the ocean’s biological pump [2]. Because of their importance, sinking particles and the composition and function of their associated community is receiving increasing interest [3–5]. Compared to larger phytoplankton, the representation of picoplankton in sinking particles has generally been thought to be negligible due to their small size, slow sinking velocity and tight regulation through grazing [6]. However, in recent years the possibility of export of small picoplankton through incorporation into aggregates or fecal pellets has been increasingly recognized [7, 8] and has been proposed to be in proportion to their total net primary production [9, 10].

The bacterial community composition of particulate material has been assessed in several different environments, suggesting that particle attached communities are phylogenetically distinct from that of the surrounding water (e.g., [5, 11–13]). Groups that are enriched on sinking particles include members of *Bacteroidetes*, *Planctomycetes*, and *Roseobacter* [3, 14]. Since particles are thought to represent diverse microscale conditions, bulk analyses of sedimented or size fractionated material may not provide high-resolution insights into the associated communities of individual particles. In addition, only few studies have considered degradation dynamics during sample collection [15]. Thus, molecular characterization of individual particles collected during short time scales may provide novel insights into the composition of sinking particles.

The North Pacific Subtropical Gyre (NPSG) is characterized by low concentrations of macronutrients and warm surface water temperatures. The ecosystem is dominated by relatively low rates of primary production and low particulate organic carbon (POC) flux at the base of the euphotic zone [16]. Primary production in the NPSG is limited by nitrogen (N) and the dissolved dinitrogen gas (N_2_) is only available to N_2_-fixing organisms (diazotrophs), which can reduce N_2_ into bioavailable ammonium. In the NPSG, diazotrophs contribute to a significant portion (26–47% during the years 2006–2013) of particulate nitrogen export [17]. However, the fate of the newly fixed N_2_ in the marine food web is not fully understood and the extent to which cells of dead or living N_2_-fixing organisms are exported from the euphotic zone on sinking particles may vary among the diverse species [18, 19]. In the oligotrophic open ocean, significant diazotrophs associated with larger size fractions of plankton include the widely distributed filamentous N_2_-fixing cyanobacterium *Trichodesmium* [20] and heterocystous-cyanobacterial endosymbionts of diatoms [21]. While diatoms can be exported directly due to their relatively heavy silicified cell walls [22] *Trichodesmium* has been suggested to have low direct export efficiency [23]. Small unicellular cyanobacterial diazotrophs (UCYN), are also highly prevalent (e.g., [24]) and may consequently play a significant role in POC export, but little is known about their possible export mechanisms.

N_2_ fixation is an energy demanding and oxygen (O_2_) sensitive process. Cyanobacterial diazotrophs have various mechanisms for protecting the nitrogenase enzyme from inactivation by O_2_ including temporal separation of N_2_ fixation and photosynthesis, heterocyst formation, and symbiotic interactions. The unicellular cyanobacterial diazotrophs (UCYN-A, UCYN-B and UCYN-C) exhibit a range of cellular strategies and interactions [25]. UCYN-A lives in association with a photosynthetic picoeukaryote, while UCYN-B (*Crocosphaera* sp.) can be found free-living but may also form colonies, aggregates or live in symbiosis with a diatom [26]. The physiology of the broadly defined UCYN-C group is still largely unknown. To explore diazotroph diversity, the *nifH* gene, a gene encoding a subunit of the nitrogenase enzyme, has been frequently used. In recent years *nifH* gene sequences related to non-cyanobacterial, heterotrophic diazotrophs have been reported from various oligotrophic ocean regions [27, 28]. How heterotrophic diazotrophs support N_2_ fixation in the oxygenated surface ocean is not yet understood and the significance to global N_2_ fixation rates is debated [29, 30]. It has been proposed that heterotrophic N_2_ fixation may take place in close association with phytoplankton or within interiors of particles but this has not yet been resolved [29].

Sinking particles constitute a diverse spectrum of environments for their colonizers based on the history of the organic matter by which they are formed. Low O_2_ concentrations within sinking particles and aggregates due to microbial remineralization of organic matter have previously been reported [31, 32]; these microenvironments would be potential niches for O_2_-sensitive diazotrophs. In this study, we investigate the bacterial composition and the associated populations of diazotrophs on both individually picked and bulk samples of sinking particles in the NPSG, and compare the community composition with that of the surrounding seawater. Using high-throughput *nifH* gene amplicon sequencing we also investigate whether diazotroph biomass is exported on sinking particles and if particles could be potential loci for heterotrophic N_2_ fixation in the oligotrophic ocean.

## Methods

### Sample collection at sea

**Fig. 1:**
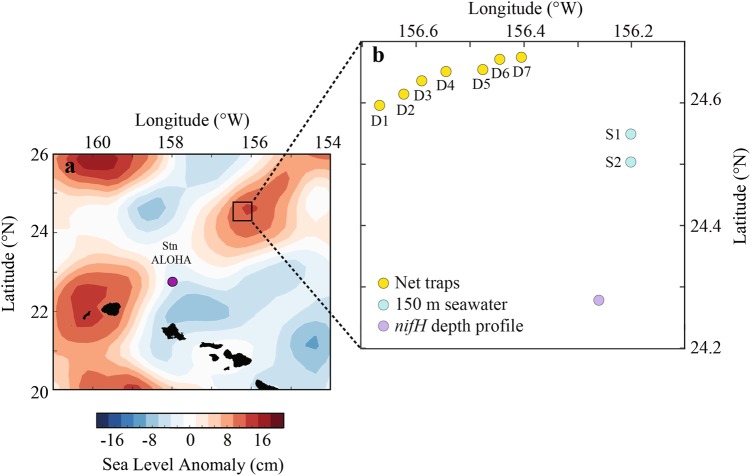
Map showing the sampling location of the net traps (D1–D7) which were collected within a total distance of 16 km, 150 m seawater samples (S1 and S2) and vertical depth profile collected for *nifH* community analysis located in an anticyclonic eddy north of Hawaii adapted from Wilson et al. [50]. **a** Patterns of sea level anomaly. **b** Sampling locations

Lagrangian sampling was performed during the HOE-Legacy 2B cruise (KOK1507) in the NPSG onboard the R/V Ka’imikai-O-Kanaloa in an anticyclonic eddy (Fig. 1). Water column properties (0–200 m) were determined from vertical depth profiles using a rosette of Niskin bottles equipped with a fluorometer and temperature, conductivity and oxygen sensors every 4 h during 25 July to 3 August 2015. Surface-tethered particle net traps with a diameter of 1.25 m (1.23 m^2^ area of collection) and a 20 µm mesh cod end [33] were deployed at 150 m for ~5–6 h. A total of 7 deployments (Fig. 1, D1–D7) were conducted over a 48 h period (Table S1). An acoustic release was used to close the traps prior to recovery so that material from the overlying water column was not inadvertently sampled. Traps were recovered immediately upon closure, except in the case of net trap D4, which was closed after ~6 h but could not be recovered until ~13 h after it was deployed (Table S1). Particle material was divided in acid-rinsed 500 ml HDPE bottles as described in Collins et al. [34] and processed immediately. Bulk particle samples were obtained by filtering 10 ml of the material collected in the traps through a 0.2 µm pore size Supor filter (Pall Corporation, New York, NY, USA). The filters were placed in cryo tubes and immediately frozen in liquid N.

### Sampling of individual particles and imaging

Individual particles (220 in total; ~20–500 μm in diameter) were visualized using a Nikon AZ100 microscope and a Nikon NI-150 illuminator and images were acquired using a Nikon DS-Fi1 camera. Individual particles were handpicked using a micropipette and placed in PCR tubes. The pipette was rinsed with 2 µl sterile filtered 150 m seawater and the rinse was placed together with the particle in the tube. The tubes were frozen at −80 °C. In parallel, individual particles were placed on glass slides, fixed in 2% paraformaldehyde and mounted using ProLong Diamond with DAPI (Molecular probes, Eugene, OR, USA). Images of DAPI stained particles and chlorophyll a containing cells were obtained using a Zeiss Axioplan 2 and a Zeiss AxioCam HRc camera and the software Zeiss AxioVision Rel.4.8. The filter cube for the chlorophyll a detection was a Zeiss high efficiency filter set 09, with excitation BP at 450–490 nm and emission at LP515 nm.

To allow for a comparison between the particle samples and the surrounding seawater, seawater samples were collected from 150 m (Fig. 1, S1 and S2). Seawater (2 l) was filtered gently through a 10 μM polycarbonate filter (Sterlitech, Kent, WA, USA) or a 3 μM polycarbonate filter (Sterlitech, Kent, WA, USA) and subsequently though a 0.2 μM Supor filter in 25 mm Swinnex filter holders (Millipore, Billerca, MA, USA) using a peristaltic pump. Samples (2 l) were also collected from a vertical seawater profile (5, 15, 25, 35, 45, 60, 75, and 100 m; Fig. 1) on 0.2 μM Supor filters to allow for analysis of diazotroph community composition. The filters were placed in sterile 1.5 ml cryovials containing 0.1 g autoclaved glass beads (BioSpec, Bartlesville, OK, USA), immediately flash frozen in liquid N and stored at −80 °C until DNA extraction.

### DNA extraction and particle lysis

DNA was extracted from seawater and bulk particle samples using the Qiagen Plant Minikit and QIAcube (Qiagen, Valencia, CA, USA). The filters were thawed at room temperature and 400 µl AP1 buffer was added to the sample tubes. The samples were freeze-thawed three times using liquid N and a 65 °C water bath and shaken at full speed for 2 min in a FastPrep-24 bead beater (MP Biomedicals, Irvine, CA, USA). The samples were Proteinase K treated (45 µl of 20 mg ml^−1^ Proteinase K) for 1 h at 55 °C with moderate shaking and RNase A treated for 10 min at 65 °C. The filters were removed from the sample tubes using sterile needles and the further extraction steps were carried out according to the manufacturer’s protocol. Samples of individual particles were prepared for PCR amplification by subjecting them to three freeze-thaw cycles using liquid N and a 65 °C water bath, adding Lyse and Go PCR Reagent (10 µl; Thermo Scientific, Rockford, IL, USA) and placing the tubes in a thermocycler with a program of 30 s at 65 °C, 30 s at 8 °C, 90 s at 65 °C, 180 s at 97 °C, 60 s at 8 °C, 180 s at 65 °C, 60 s at 97 °C, 60 s at 65 °C and an 80 °C hold while the PCR amplification mixtures were set up.

### 16S rRNA and *nifH* gene amplicon sequencing and sequence analysis

A targeted amplicon sequencing (TAS) approach as described by Green et al. [35] was used to amplify the V3-V4 variable region of the 16S rRNA gene with the CS1_341F (5′-ACACTGACGACATGGTTCTACACCTACGGGNGGCWGCAG-3′) and CS2_806R (5′-TACGGTAGCAGAGACTTGGTCTGACTACHVGGGTATCTAATCC-3′) primers. The PCR was performed in 25 µl reaction volumes with 1× PCR buffer, 2.5 mM MgCl_2_, 200 µM dNTPs, 0.25 µM of each primer and 0.3 µl Invitrogen Platinum Taq (Invitrogen, Carlsbad, CA, USA). The PCR conditions were 5 min denaturation at 95 °C, followed by 25 cycles of 40 s denaturation at 95 °C, annealing for 40 s at 53 °C and elongation for 60 s at 72 °C, and ending with a final elongation for 7 min at 72 °C. For amplification of the *nifH* gene, a nested PCR protocol was performed using the nifH3 and nifH4 primers in the first PCR followed by a second amplification with nifH1 and nifH2 primers [36] with CS1 and CS2 linkers [37]. For the PCR reaction mixtures 1× PCR buffer, 4 mM MgCl_2_, 200 µM dNTPs, 0.5 µM of each primer and 0.3 µl Invitrogen Platinum Taq (Invitrogen) were used. The PCR conditions were 3 min denaturation at 95 °C, followed by 25 cycles in the first amplification and 30 cycles in the second PCR of 30 s denaturation at 95 °C, annealing for 30 s at 55 °C for the first amplification and 57 °C for the second PCR and elongation for 45 s at 72 °C, and ending with a final elongation for 7 min at 72 °C.

For the seawater and bulk particle samples, 1 µl of the DNA extract was used as a template and PCR reactions were run in triplicates. For the individual particles, 2.5 µl of the lysed solution was used as a template. Negative extraction controls (blank filter) and negative PCR controls (only water instead of template) were included and did not result in any visible amplification when 5 µl amplification product was loaded onto a 1.4% agarose gel. The amplicons were submitted for sequencing to the DNA Services (DNAS) Facility at the University of Illinois at Chicago. At the DNAS Facility an additional PCR amplification was performed to incorporate barcodes and sequencing adaptors to the final amplicons. The sequencing was performed on an Illumina MiSeq sequencer using standard V3 chemistry with paired-end, 300 bp long reads and demultiplexing of reads was performed on instrument.

For the 16S rRNA gene libraries, the forward and reverse FASTQ files were merged using PEAR [38]. To obtain high-quality data, the sequences were trimmed using a quality threshold of *p* = 0.01 in the software package CLC genomics workbench (v7; CLC Bio, Qiagen, Boston, MA, USA) and sequences that were less than 350 bp in length and lacked either primer were discarded. Chimeric sequences were identified using the USEARCH [39] algorithm as compared with the GreenGenes 13.8 database [40] and filtered from the dataset. De novo operational taxonomic unit (OTU) clusters (97% sequence similarity) were generated, taxonomy of the OTU centroid sequence was assigned using UCLUST in the QIIME v1.8 pipeline [41] and singletons were removed. Representative sequences for each OTU were generated and closest relatives were identified using Blastn. The libraries were rarefied to 15 000 sequence depth and samples with <15,000 sequences were removed. The number of observed OTUs, Shannon H and Chao1 diversity indices were calculated using Explicet [42]. A Bray–Curtis similarity matrix was calculated using the software package Primer6 (Primer-E). To identify OTUs whose abundance in seawater differed significantly from that in bulk particle samples based on hierarchal taxonomy, a two-sided Welch’s *t*-test with confidence intervals was used in the STAMP v2.01 software [43]. Statistical confidence intervals were calculated for a 95% nominal coverage. PERMANOVA analyses were done using the vegan package in R, and function adonis using 999 permutations.

The paired-end FASTQ files from the *nifH* amplicon sequencing were merged using the software package PEAR [38]. The libraries were quality filtered (phred20) using the QIIME pipeline [41]. A de novo chimera check and OTU clustering (97% similarity) was done using USEARCH61 [39], representative sequences were generated and singletons were removed from the dataset. The sequences were translated and aligned using MEGA6.06 [44]. OTUs with representative sequences containing stop codons or frameshifts were removed from the dataset, corresponding to <9% of the total number of sequence reads. To identify UCYN-A sublineages, all UCYN-A reads were extracted from the dataset by clustering all reads (post-chimera check) at 95% nucleotide (nt) identity after adding representative UCYN-A sequences. Oligotypes were determined using the oligotyping pipeline created by Eren et al. [45] using the same entropy positions determined in Turk-Kubo et al. [46]. Representative *nifH* sequences were annotated based on *nifH* cluster classification [47] according to the recently described CART model [48] complemented by a phylogenetic tree analysis to identify groups of cyanobacterial diazotrophs. The sequences have been submitted to NCBI Sequence Read Archive (SRA) with accession number SRP111426.

### Abundance of diazotrophs

To enumerate diazotroph groups in the vertical profile, quantitative polymerase chain reaction (qPCR) targeting UCYN-A1, UCYN-B, *Trichodesmium* spp., and three diatom–diazotroph associations (Het-1, Het-2, and Het-3) was used and conducted as previously described [49].

## Results

### Water column features in the sampling area

**Fig. 2:**
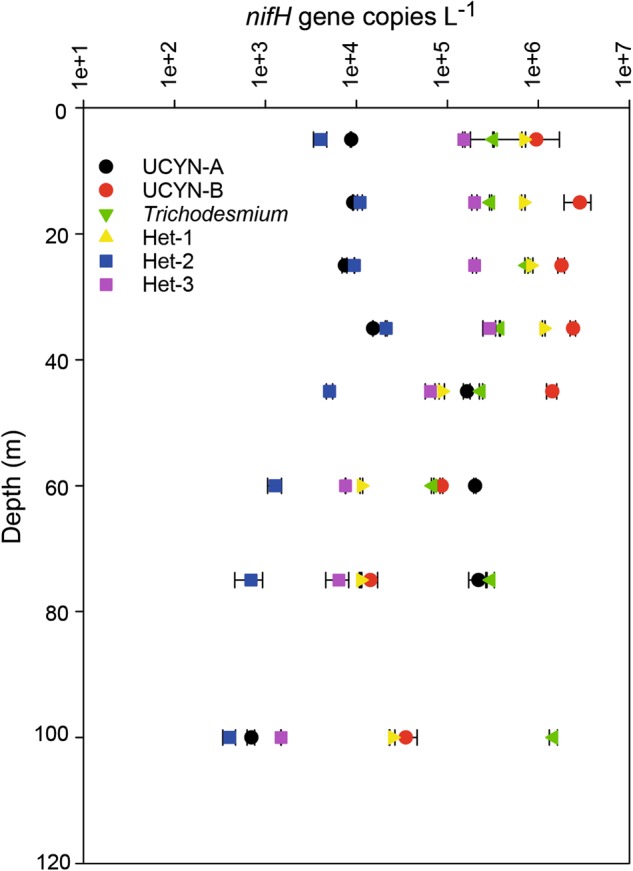
Average *nifH* gene abundances for target groups measured using qPCR in a vertical profile. Error bars indicate standard deviations based on triplicate measurements

The area of sampling was characterized by an anticyclonic eddy feature coinciding with elevated chlorophyll a and productivity (described in ref. [50]; Fig. 1). The mixed layer depth averaged around 30 m and the deep chlorophyll a maximum varied between 120 and 130 m. The surface photoautotroph population was dominated by *Prochlorococcus* at 1.6 × 10^8^ cells l^−1^. *Synechococcus* and photosynthetic picoeukaryotes averaged 1.1 × 10^6^ and 0.4 × 10^6^ cells l^−1^, respectively [50]. The abundances of key diazotroph groups were determined, indicating that *Crocosphaera* sp. (UCYN-B) was present in unusually high abundances in the upper 50 m (average 1.9 × 10^6^ *nifH* gene copies l^−1^; Fig. 2). UCYN-A1, *Trichodesmium* spp., and three diatom-diazotroph associations (Het-1, Het-2, and Het-3) were also detected throughout the vertical profile (5–100 m) albeit at lower abundances (Fig. 2).

### Microscopic analysis of particles

**Fig. 3:**
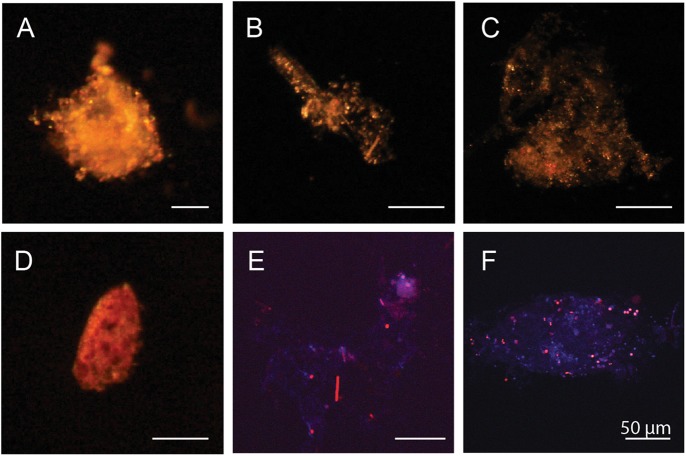
Microscopy images showing examples of individual particles from particle samples collected from net traps at 150 m. **a**–**d** The particulate material ranged from partly decayed planktonic organisms to loosely associated aggregates and fecal pellets. **e**, **f** DAPI stained particles showing that the particles were associated with both chlorophyll a containing cells (red) and non-pigmented cells (blue)

Sinking particles were visualized and picked individually under a dissection light microscope. Many of the particles appeared to be loosely associated material, but fecal pellets and aggregates with a more dense appearance were also observed (Fig. 3). Filaments of *Trichodesmium* were observed in material from the net traps D1, D4, D5, and D6. Epifluorescence microscopy showed that the particles were heavily colonized by photosynthetic (chlorophyll a containing) and non-photosynthetic bacteria of various sizes (Fig. 3).

### 16S rRNA gene composition on particles

**Fig. 4:**
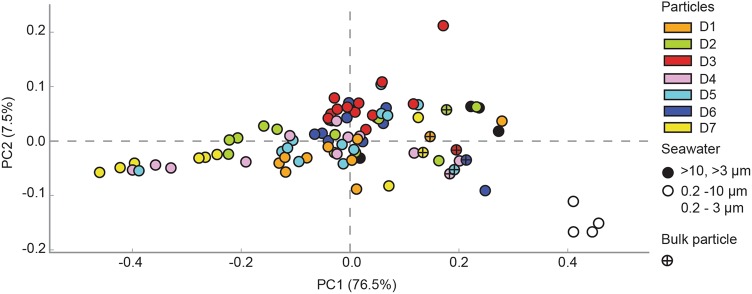
Principal component analysis (PCA) plot calculated using the 16S rRNA gene class level of the taxonomical hierarchy for each sample. The two first principal components are plotted with the proportion of variance explained by each component indicated in brackets. The numbers of sequences were normalized to the total number of sequences in each library and unclassified sequences were retained. Each sample is represented by a circle, with a color indicating the net trap number or the fraction of the seawater. Bulk particle samples for each net trap are labelled with a (+) in the circle

The bacterial composition of the bulk particle samples, individual particles and seawater samples from the depth of deployment (150 m) was investigated. In total, >2 million high quality 16S rRNA gene sequences were analyzed (Supplementary Table S2). The lowest diversity was seen for the individual particles where the number of OTUs varied between 387 and 875 OTUs. The highest diversity of the bacterial community was observed in the seawater samples (up to 1706 OTUs; Supplementary Figure S1). Principal coordinate analysis showed that the bacterial community composition of the particles on both the class and OTU level was most similar to the >10 and >3 µm seawater samples; Fig. 4 and Supplementary Figure S2). The bulk particle samples exhibited less variation in composition compared to the individual particles and were significantly different from the 0.2–10 and 0.2–3 µm seawater samples (PERMANOVA, *p* = 0.005; Fig. 4 and Supplementary Figures S2 and S3). The individual particles were highly variable in composition but a few particles collected from the same net trap had relatively high similarity (>75% Bray–Curtis similarity; Supplementary Figure S3).

**Fig. 5:**
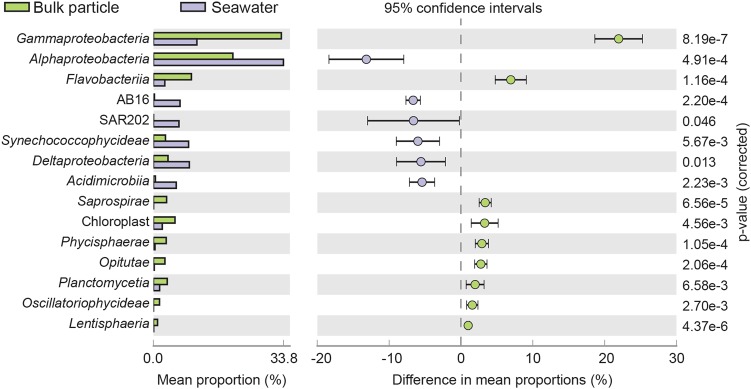
Bar plot showing the mean proportion and the difference in mean proportion (16S rRNA gene) between the respective groups of significantly enriched (*p* < 0.05; Welch’s *t*-test) classes with a difference in mean proportions of >1%. Error bars indicate standard deviation and corrected *p*-values are indicated to the right. The small size fraction seawater samples (0.2–10 and 0.2–3 µm) are shown in purple, and bulk particle samples from each net trap (D1–D7) are shown in green

To identify groups whose abundance differed significantly between the small size fraction of the seawater samples (0.2–10 and 0.2–3 µm) and the bulk particle samples, a group comparison between the mean proportions of sequences per sample was done both at class and OTU level. The particle sequence libraries were dominated by *Gammaproteobacteria* (Fig. 5), including *Alteromonadales, Vibrionales* and *Oceanospirillales*. *Bacteroidetes* was the second most abundant phylum, and *Flavobacteria* were significantly (*p* < 0.001; Welch’s *t*-test) enriched in the particle samples compared to the small size fraction seawater samples (Fig. 5). At the fine-scale OTU level, 24 OTUs with >1% difference in mean proportion were identified (Table 1). Typically free-living genera such as *Synechoccoccus*, *Prochlorococcus* and *Pelagibacter* were significantly (*p* < 0.01; Welch’s *t*-test) enriched in the seawater samples compared to the bulk particle samples while OTUs within the order Alteromonadales (*Alteromonas;* OTU_4074 and *Pseudoalteromonas;* OTU_26436) were significantly (*p* < 0.01; Welch’s *t*-test) enriched on particles (Table 1).

**Table 1:** Affiliation and closest relatives in Genbank to the enriched (>1% difference in mean proportions) 16S rRNA gene OTUs in bulk particle samples (D1–D7) compared to seawater samples (0.2–10 and 0.2–3 µm)

OTU ID	Class affiliation	Closest relative	Similarity	Accession number
Enriched in bulk particle samples				
OTU_43683	*Alphaproteobacteria*	*Shimia sp*.	100%	KT720466.1
OTU_20532	*Alphaproteobacteria*	*Tropicibacter multivorans*	100%	KP843700.1
OTU_16894	Chloroplast	*Rhizosolenia*	97%	KJ958482.1
OTU_25878	*Flavobacteriia*	Unclassified *Bacteroidetes*; *Flavobacteriales*		
OTU_4074	*Gammaproteobacteria*	*Alteromonas sp*.	100%	KX356439.1
OTU_26436	*Gammaproteobacteria*	*Pseudoalteromonas sp*.	100%	KX806641.1
OTU_22018	*Gammaproteobacteria*	Unclassified *Gammaproteobacteria*; *Oceanospirillales*		
OTU_29878	*Gammaproteobacteria*	*Thalassolituus oleivorans*	97%	KF170318.1
OTU_18280	*Gammaproteobacteria*	*Photobacterium angustum*	100%	KC534344.1
OTU_44280	*Opitutae*	Uncultured *Verrucomicrobia*; *Puniceicoccaceae*	99%	HQ675288.1
OTU_8705	*Saprospirae*	Unclassified *Bacteroidetes*; *Saprospiraceae*		
Enriched in free-living seawater samples				
OTU_19700	*Acidimicrobiia*	Uncultured *Actinobacteria*	100%	HQ675198.1
OTU_36708	*Alphaproteobacteria*	*Pelagibacteraceae*	100%	HQ675347.1
OTU_39648	*Alphaproteobacteria*	*Pelagibacter sp*.	100%	LN850157
OTU_30217	*Alphaproteobacteria*	*Pelagibacteraceae*	99%	JF488446.1
OTU_817	*Alphaproteobacteria*	Uncultured *Alphaproteobacteria*	97%	JF488548.1
OTU_18321	*Alphaproteobacteria*	*Pelagibacteraceae*	99%	HQ675689.1
OTU_26273	*Deltaproteobacteria*	Uncultured *Deltaproteobacteria*; Sva0853	99%	U65908.1
OTU_6835	*Deltaproteobacteria*	Uncultured *Deltaproteobacteria*; Sva0853	100%	HQ675271.1
OTU_31137	SAR202	Unclassified *Chloroflexi*		
OTU_15929	AB16	Uncultured SAR406	96%	JF488613.1
OTU_23515	*Synechococcophycideae*	*Prochlorococcus*	100%	CP007753.1
OTU_23585	*Synechococcophycideae*	*Synechococcus sp*.	100%	JF306716.1

**Fig. 6:**
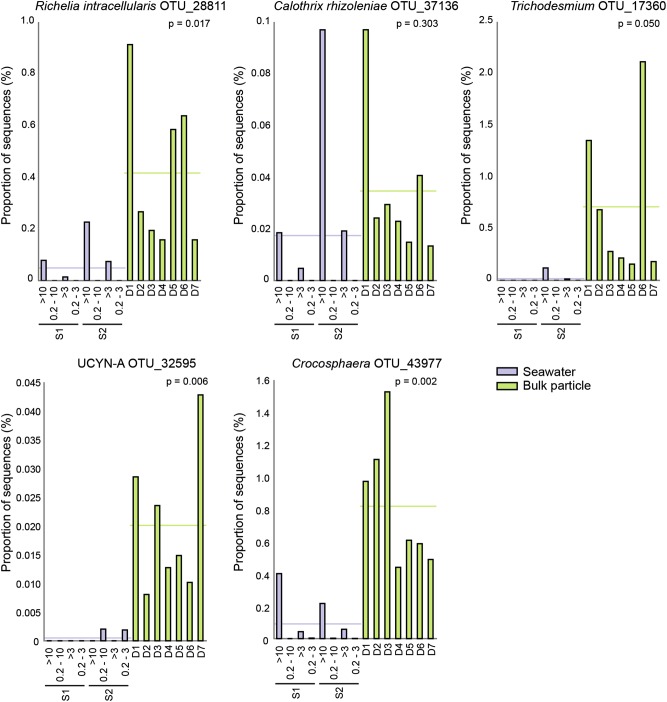
Group comparison of the sequence library proportions of OTUs related to the 16S rRNA genes of known diazotrophs (>97% identity) in seawater and bulk particle samples. The bar plots show the proportion of sequences for each sample. The seawater samples (S1 and S2; >10, 0.2–10, >3, and 0.2–3 µm) are shown in purple and the bulk particle samples from each net trap (D1–D7) are shown in green. The horizontal line in each graph shows the mean proportions of sequences for the respective sample groups and p-values are given for each graph

The relative abundance of chloroplast sequences was significantly higher in the bulk particle samples compared to the seawater samples (*p* < 0.001; Welch’s *t*-test) and the most frequently detected chloroplast sequences were Stramenopiles (2.0 ± 2.2% mean sequence proportion) and Haptophycea (0.5 ± 0.3% mean sequence proportion; data not shown). Notably, the chloroplast sequences of *Rhizosolenia imbricate* (OTU_16894), a diatom which has been reported to contain the endosymbiotic N_2_-fixing bacterium *Richelia intracellularis* [51], were among the most highly enriched OTUs in the bulk particle samples (Fig. 6 and Table 1). In addition, OTUs closely related (>97% identity) to the 16S rRNA gene sequences of the diazotrophs *Crocosphaera*, *Trichodesmium* and UCYN-A were consistently present and significantly enriched in the bulk particle samples compared to the seawater samples (Fig. 6).

### Presence and composition of diazotrophs on particles

To identify the diazotrophs present on particles the *nifH* gene was amplified from bulk particle and individual particle samples. All of the bulk particle samples had strong *nifH* gene amplicons, visualized through gel electrophoresis. For the individual particles, *nifH* genes amplified from 49 out of the 76 individual particles (Supplementary Tables S1 and S2). For the particles sampled from net traps D3 and D6, all resulted in *nifH* amplicons while for particles from net traps D1 and D2 only <30% of the investigated particles amplified.

In total, >4.1 million high quality *nifH* sequences in 511 OTUs (97% amino acid similarity clustering) were analyzed (Supplementary Table S2). Classification of the *nifH* OTUs using a tree-placement approach showed that 44.5% of sequences in bulk particle samples were *nifH* Cluster 1B Cyanobacteria (defined in [47]) with representative OTUs from endosymbionts of diatoms (Het-1, Het-2 and Het-3), *Trichodesmium*, UCYN-A, *Crocosphaera* (UCYN-B) and *Cyanothece* (UCYN-C) (Supplementary Table S3). The proportion of *nifH* Cluster 1B was lower in the bulk particle samples compared to the vertical profile where *Trichodesmium*, UCYN-A and UCYN-B were the most prominent 1B phylotypes. The UCYN-A sequences in the bulk particle samples consisted primarily of the UCYN-A sublineages UCYN-A1 and UCYN-A3. The proportion of UCYN-A3 sequences was consistently higher compared to UCYN-A1 which has previously been reported as the dominant lineage in the NPSG water column ([46]; Supplementary Figure S4). The *nifH* Cluster IG, a *nifH* cluster comprised primarily of gammaproteobacterial phylotypes was represented by 50.3% of the bulk particle sequences (Supplementary Table S3). Sequences affiliating with *nifH* Cluster III, composed of diverse sequences mostly from anaerobic bacteria and archaea, Cluster 1P, which includes *Gamma-* and *Betaproteobacteria* phylotypes, and Cluster 1J/1K, which includes *Alpha*- and *Betaproteobacteria* were consistently recovered at a higher relative abundances in the bulk particle samples compared to the vertical profile (Supplementary Table S3).

**Fig. 7:**
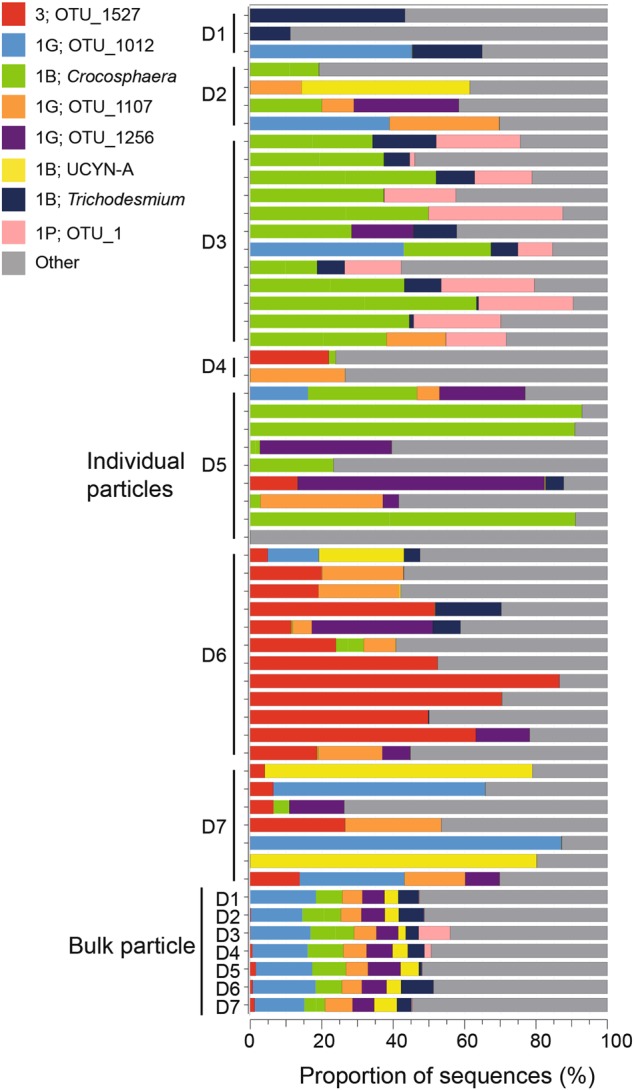
Bar plot showing the proportion of *nifH* sequences of the nine most abundant OTUs (representing ~60% of the total *nifH* sequences in the dataset) for each of the individual particles and bulk particle samples from each net trap (D1–D7). The legend indicates the *nifH* cluster affiliation and the OTU ID for each of the OTUs

The *nifH* composition of the bulk particle samples was highly consistent during the 48 h sampling, (76.6–90.5% Bray–Curtis similarity) while large variations between individual particles were observed (Fig. 7 and Supplementary Figure S5). The composition of individual particles was highly dependent on the net trap sample from which the particles were collected. For example, *Crocosphaera* (UCYN-B; Cluster 1B) was most prevalent in individual particles collected from the D3 and D5 traps (Fig. 7). Similarly, OTU_1527 (Cluster III) which had 96% nt identity to a sequence reported from Seto Inland Sea in Japan (Accession number LC063947; [52]) and 99% amino acid identity to *Desulfovibrio putealis* (Accession number WP_027192250) was the dominating OTU in particles collected from net trap D6 (up to 87 %). The OTU_1 (Cluster 1 P), affiliating with the Betaproteobacteria *Azoarcus sp*. and *Dechloromonas aromatica* (<88% nt identity), was most frequently detected in individual particles collected from net trap D3 (Fig. 7). Three gammaproteobacterial OTUs were represented among the eight most abundant OTUs including OTU_1107, representing the widespread gammaproteobacterial group γ24774A11 (98% nt identity; [28, 53]). However, although the gammaproteobacterial OTUs represented a large proportion of sequences in the bulk particle samples, their presence in individual particles was highly variable (Fig. 7 and Supplementary Table S3).

## Discussion

Large diameter net traps allow for collection of sinking particles over comparatively short timescales (4–7 h). Over the time course spanning 48 h, when our seven net traps were deployed and collected, the community composition of the bulk particle samples were relatively similar to one another (Fig. 4 and Supplementary Figure S2). This suggests that there is a temporal consistency in the composition of the microbial community on sinking particles over diel timescales. Moreover, the bulk particle samples in this study were enriched in taxa known to be found in association with particles and the composition was similar to that recently reported for traps deployed at various depths at Station ALOHA in the NPSG [3]. By contrast, the sequence libraries of individually picked particles (~50–200 µm in diameter) showed large variations between the samples indicating that the individual particles are inhabited by specialized bacterial taxa and offer distinct ecological niches. Notably, the individual particles collected over the size range and characteristics that could be easily distinguished and picked using a micropipette were not representative of the bulk particle samples (Fig. 4 and Supplementary Figure S2). This potentially underscores the importance of small particles for carbon flux [54].

The 16S rRNA gene libraries indicated that the particles contained both large and small size classes of cyanobacterial diazotrophs. This was further confirmed by amplification and sequencing of the *nifH* gene in a majority of the individual particles. Our results demonstrate that in addition to diazotrophs associated with larger cells such as the diatom-*Richelia* symbiosis [19] typically associated with sinking particles, unicellular cyanobacterial diazotrophs were also contributing to direct POM export. Unicellular cyanobacterial diazotrophs have previously been visualized in association with inert particles and in association with eukaryotic cells [55, 56]. However, the cellular strategies of unicellular cyanobacterial diazotrophs and whether they contribute to N_2_ fixation rates in both the free-living and particulate fractions has not yet been resolved. Why cyanobacterial diazotrophs of diverse size classes are found in association with sinking particles—a process that ultimately removes them from the euphotic zone—and whether these diazotrophs are active on particles have yet to be determined.

The presence of unicellular cyanobacterial diazotrophs on particles has implications for the particular lifestyles of specific diazotroph species as some may be more prone to aggregation or grazing than others. At the time of sampling the unicellular diazotroph *Crocosphaera* was present at unusually high abundances which likely reflected their presence in collected bulk particle samples. Some strains of *Crocosphaera* are known to produce extracellular polysaccharides [57, 58] which can contribute to particle formation and thereby they may have a direct role in carbon export [59]. The widely distributed, uncultivated, symbiotic N_2_-fixing cyanobacterium UCYN-A has distinct co-occurring sublineages, and in the NPSG, the two commonly occurring sublineages are UCYN-A1 and UCYN-A3 [46, 60]. While the symbiotic partner of UCYN-A3 has not yet been identified or visualized, UCYN-A1 lives in association with a small haptophyte related to *Braarudosphaera bigelowii* and the size of the cell consortia is 1–3 µm in diameter [60]. The enrichment of UCYN-A3 on particles suggests that these organisms aggregate on particles more efficiently or have adapted to a particle attached lifestyle. Thus, the strategies of the two closely related and co-occurring sublineages may be different.

A large proportion of the sequences in the *nifH* gene libraries in this study were from heterotrophic (non-cyanobacterial) diazotrophs. In the oligotrophic ocean, carbon substrates are sparse and are unlikely to support the demands of heterotrophic N_2_ fixation; consequently, their significance to N_2_ fixation has been debated. It has previously been hypothesized that nutrient-rich particles could support heterotrophic N_2_ fixation [29, 61]. To our knowledge, this is the first study showing that diverse non-cyanobacterial diazotrophs are present on sinking particles which suggests that particles may be loci for heterotrophic N_2_ fixation.

Particles collected from net trap D6 contained a large proportion of Cluster III diazotrophs and may therefore have exhibited conditions that favored anaerobic or microaerophilic bacteria. A central anaerobic micro-zone which can support anaerobic processes has been demonstrated in mm-large particles and aggregates [62, 63] and anaerobic or microaerophilic bacteria were recently reported in association with the particle size fraction (>3 µm) in the South China Sea [64] which suggests that bacteria with low O_2_ preferences could thrive on some particles. Cultivation studies of heterotrophic N_2_ fixation indicate that specific requirements of low O_2_ concentrations may be required for N_2_ fixation [65]. However, it was recently shown that the sulfate-reducing N_2_-fixing bacterium, *Desulfovibrio magneticus*, can grow in aerobic conditions [66]. Whether or not the particles that were picked in this study could provide the right conditions for Cluster III or other non-cyanobacterial diazotrophs to grow or fix N_2_ is unknown. Yet at times, the high proportion of anaerobic diazotrophs present within individual particles such as those from net trap D6 suggested that they might have been significant.

Sequencing of gene amplicons is associated with several biases. For example, it has been suggested that the *nifH* primers used in this study may favor amplification of the gammaproteobacterial γ24774A11 group [67], represented by OTU_1107 in this study, while the endosymbionts *Richelia* and *Calothrix* appear to be systematically underrepresented in sequence libraries [49]. Notably, in this study although represented in the 16 S rRNA gene libraries (up to 0.9%, Fig. 6), the mean proportion of endosymbiont *nifH* groups (Het-1, Het-2 and Het-3) was low compared to other groups in the bulk particle samples (5.0 ± 1.8%). This highlights that the differences in relative abundances between taxa need to be interpreted cautiously. In addition, the sequencing of a DNA fragment does not provide information of whether the cell is alive or even intact, thus from sequencing data alone it cannot be determined whether the detected diazotrophs were active on particles or not. In future studies, measurements of absolute abundances and N_2_ fixation rates will be necessary to elucidate the significance of diazotrophs on particles but this may be technically challenging because of the heterogeneous nature of the particle pool [54].

To our knowledge, this is the first study which characterizes the bacterial composition and presence of diazotrophs on individual sinking particles collected in the ocean. The study shows that the heterogeneity in composition on particles is significant and indicates that diazotrophs are directly involved in POC export in the NPSG. The consistent presence of various size classes of diazotrophs warrant further investigations, including quantification of specific species and measurement of N_2_ fixation activity on particles. Further studies on microscale habitats will provide clues about the processes occurring within sinking particles and the important consequences for POC export in the oligotrophic ocean.

## Electronic supplementary material

Supplementary Figure legends

Supplementary Figure S1

Supplementary Figure S2

Supplementary Figure S3

Supplementary Figure S4

Supplementary Figure S5

Supplementary Table S1

Supplementary Table S2

Supplementary Table S3
