# Supplementary material for: Diverse diazotrophs are present on sinking particles in the North Pacific Subtropical Gyre
Source: ISME J. 2018 Aug 16;13(1):170–82. doi: 10.1038/s41396-018-0259-x (PMC6299005; doi:10.1038/s41396-018-0259-x)
Supplement: Supplementary file 1 — Supplementary Figure legends [file 41396_2018_259_MOESM1_ESM.docx]

**Supplementary information**

**Diverse diazotrophs are present on sinking particles in the North Pacific Subtropical Gyre**

Hanna Farnelid^1,2*^, Kendra Turk-Kubo^1^, Helle Ploug^3^, Justin E. Ossolinski^4^, James R. Collins^4,5,6^, Benjamin A. S. Van Mooy^4^, Jonathan P. Zehr^1^

**Supplementary figure legends.**

**Figure S1.** Number of observed OTUs, Shannon H and Chao1 calculated for 16S rRNA gene libraries rarefied to 15 000 sequence depth. Bulk particle samples are indicated with crosses in circles.

**Figure S2.** Principal component analysis (PCA) plot calculated using the 16S rRNA OTU abundance table for seawater, bulk particle and individual particle samples. The two first principal components are plotted with the proportion of variance explained by each component indicated in brackets. The numbers of sequences were normalized to the total number of sequences in each library and unclassified sequences were retained. Each sample is represented by a circle, with a color indicating the net trap number or the fraction of the seawater. Bulk particle samples for each net trap are labelled with a (+) in the circle.

**Figure S3.** Dendrogram based on Bray-Curtis similarity between rarefied (15 000 sequences) and normalized 16S rRNA gene libraries from individual particles and bulk particle samples collected from the separate deployments (D1, D2, D3, D4, D5, D6 and D7) and size fractionated seawater (>10 µm, >3 µm, 0.2 – 10 µm, and 0.2 – 3 µm) samples.

**Figure S4.** Bar plot showing the number of UCYN-A sequences for classified UCYN-A sublineages in the bulk particle samples collected from the different deployments (D1, D2, D3, D4, D5, D6, and D7) in comparison to a vertical profile (5, 15, 25, 35, 45, 60, 75 and 100 m). The proportion of UCYN-A3 sequences to the total number of UCYN-A sequences is indicated to the right.

**Figure S5.** Principal component analysis (PCA) plot calculated using the *nifH* OTU abundance table for bulk particle samples and the vertical seawater profile. The two first principal components are plotted with the proportion of variance explained by each component indicated in brackets. The numbers of sequences were normalized to the total number of sequences in each library. Each sample is represented by a circle, with black color indicating bulk particle sample and color indicating depth in the vertical profile.
