# Supplementary figures and images for: Diverse diazotrophs are present on sinking particles in the North Pacific Subtropical Gyre
Source: ISME J. 2018 Aug 16;13(1):170–82. doi: 10.1038/s41396-018-0259-x (PMC6299005; doi:10.1038/s41396-018-0259-x)

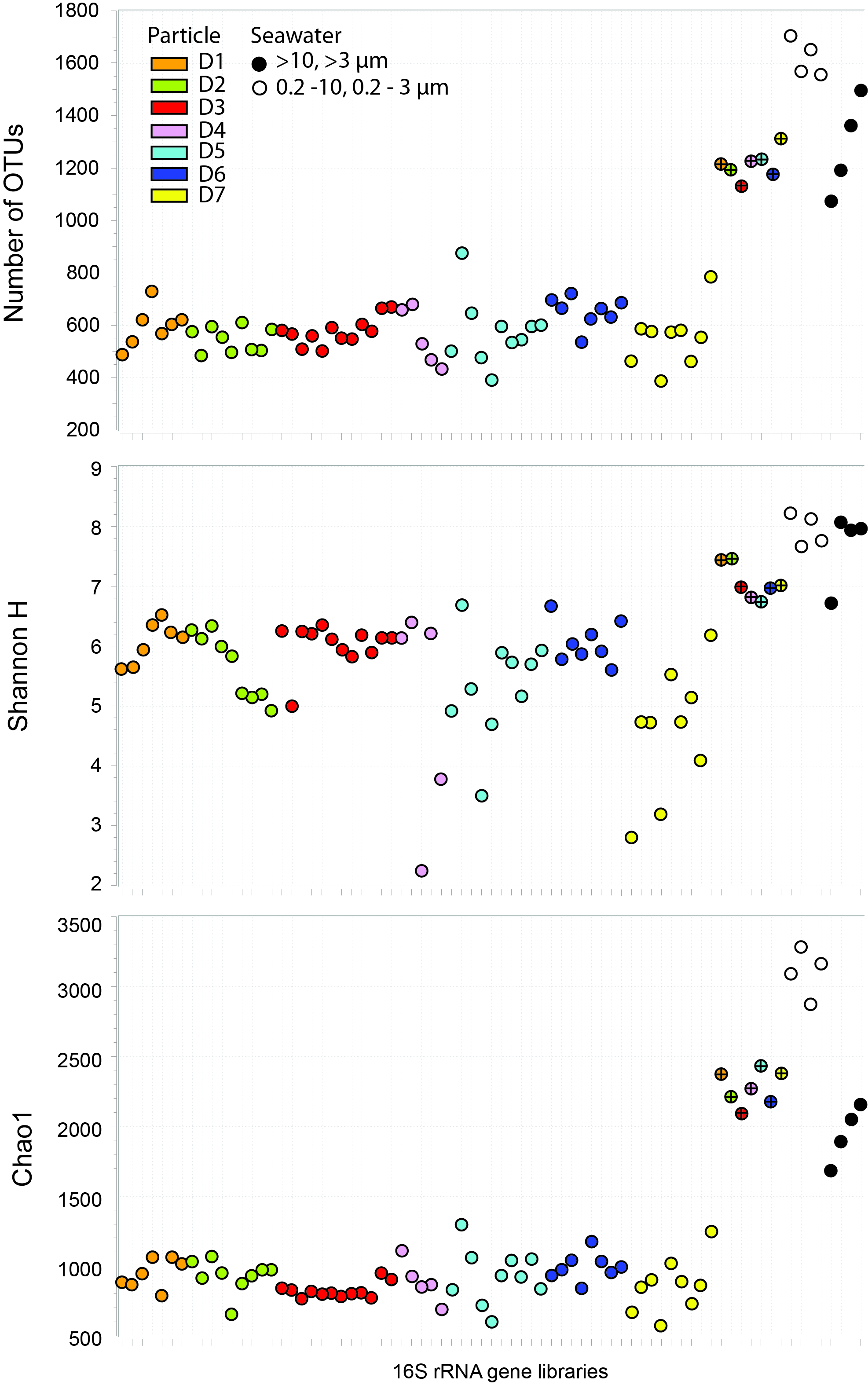

Supplement: Supplementary file 2 — Supplementary Figure S1 [file 41396_2018_259_MOESM2_ESM.tif]

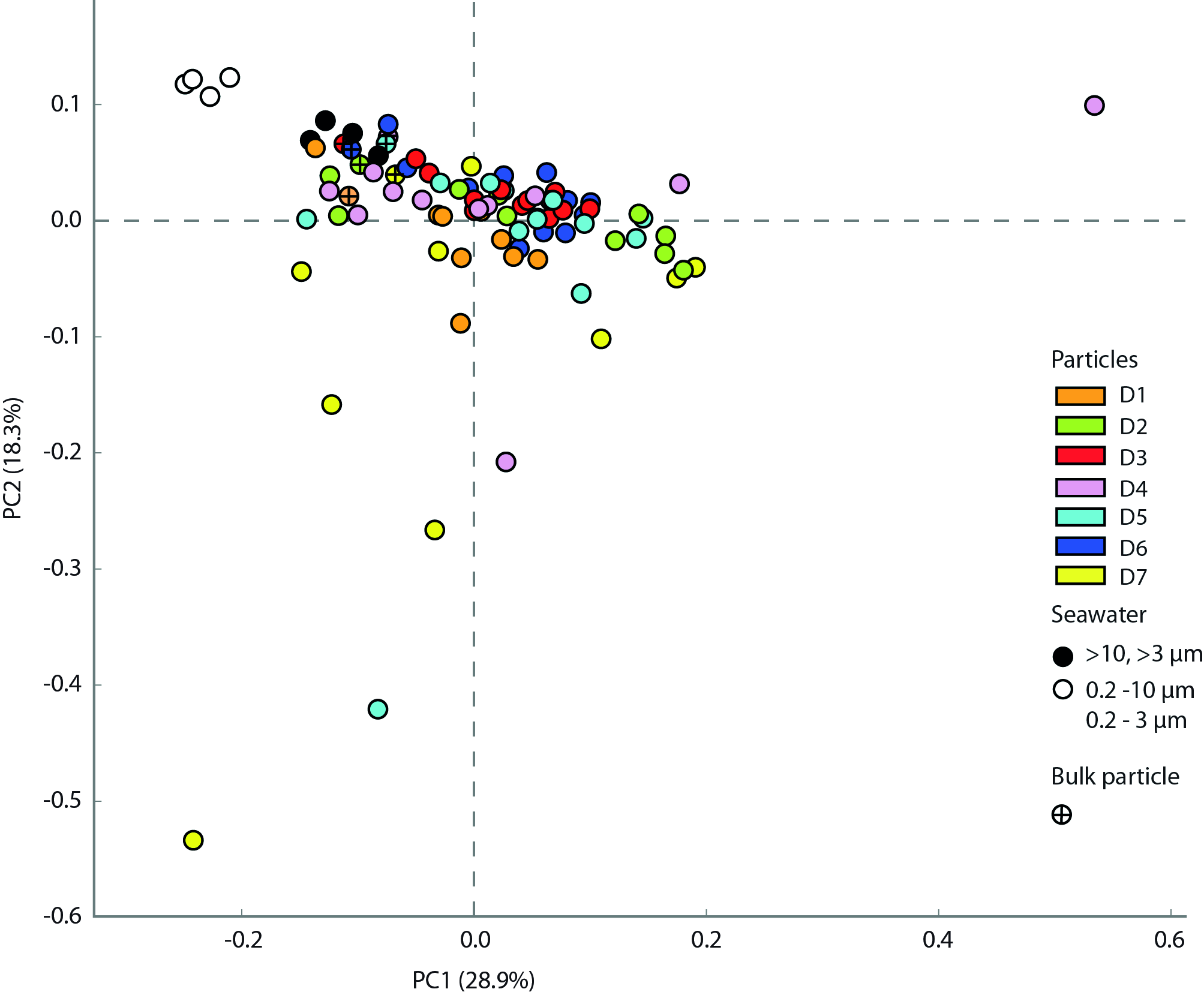

Supplement: Supplementary file 3 — Supplementary Figure S2 [file 41396_2018_259_MOESM3_ESM.tif]

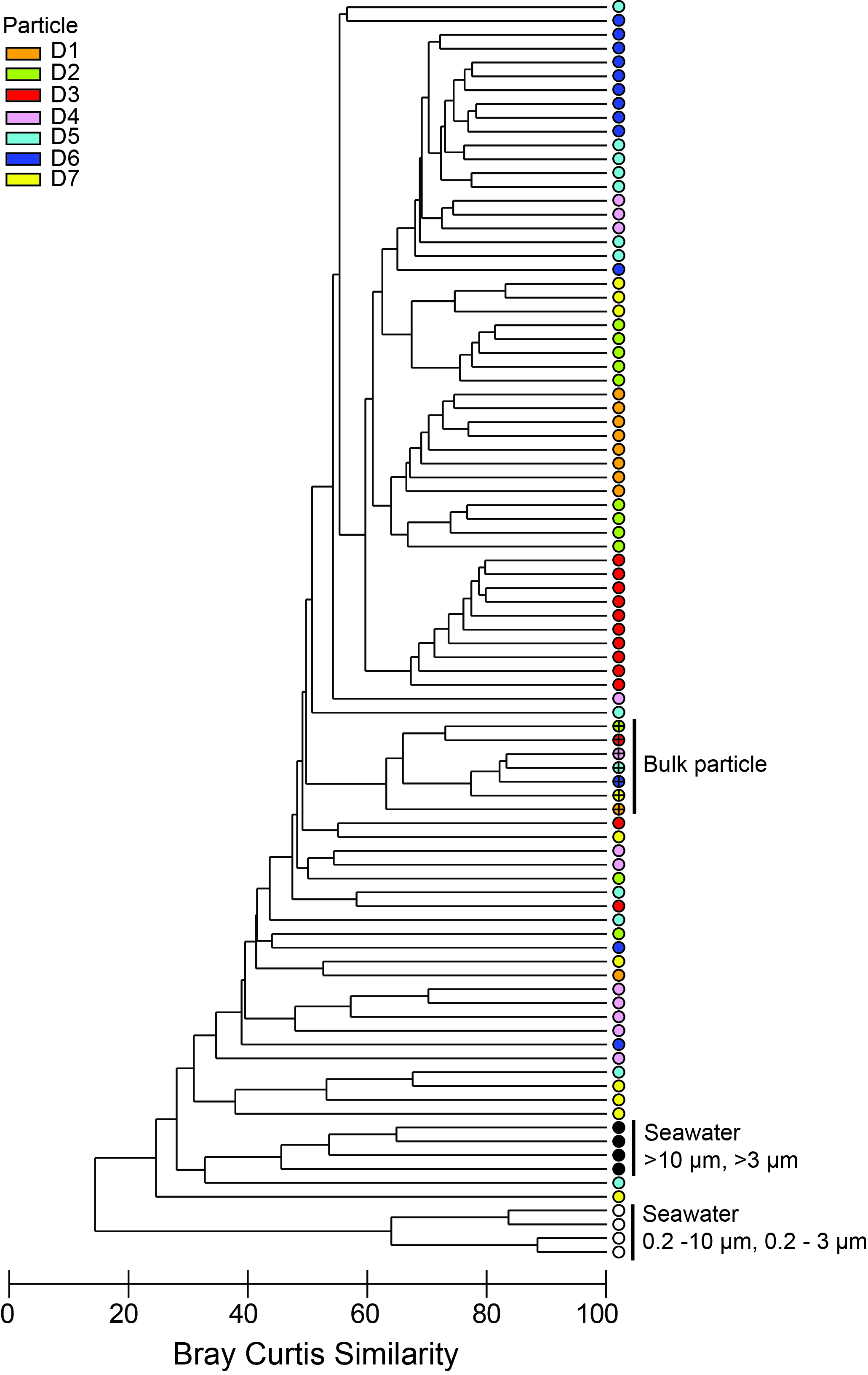

Supplement: Supplementary file 4 — Supplementary Figure S3 [file 41396_2018_259_MOESM4_ESM.tif]

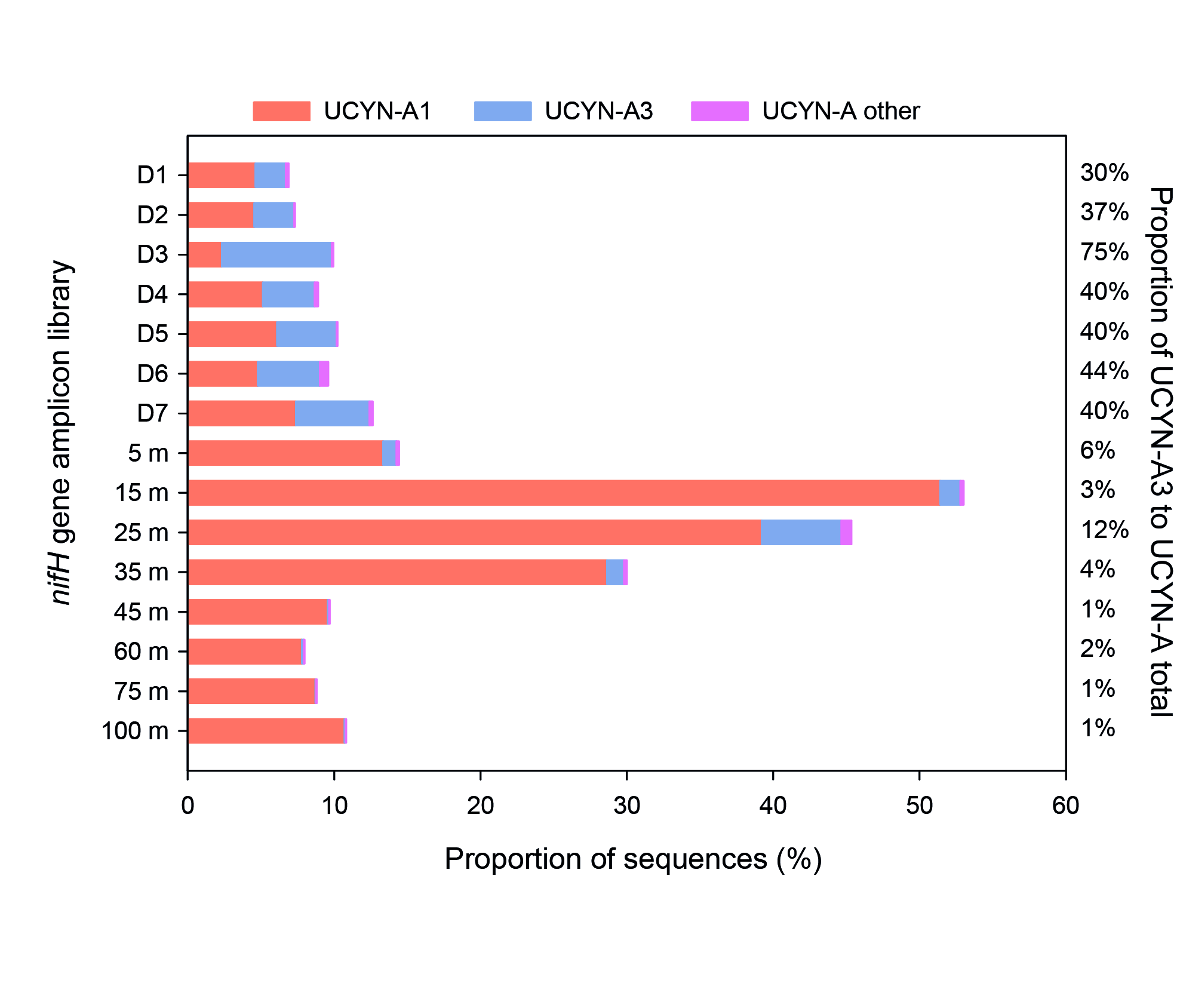

Supplement: Supplementary file 5 — Supplementary Figure S4 [file 41396_2018_259_MOESM5_ESM.tif]

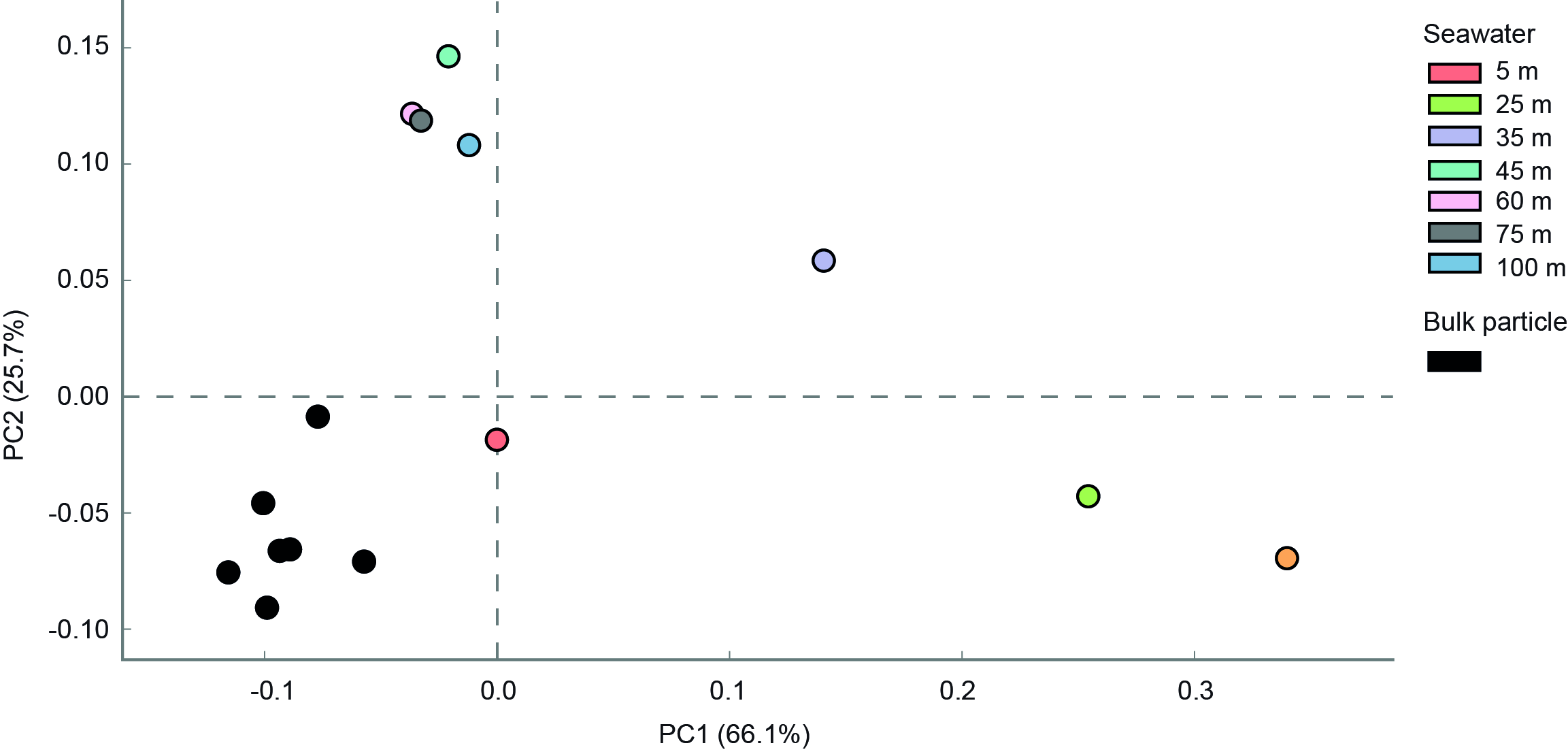

Supplement: Supplementary file 6 — Supplementary Figure S5 [file 41396_2018_259_MOESM6_ESM.tif]
