# Supplementary material for: Diverse diazotrophs are present on sinking particles in the North Pacific Subtropical Gyre
Source: ISME J. 2018 Aug 16;13(1):170–82. doi: 10.1038/s41396-018-0259-x (PMC6299005; doi:10.1038/s41396-018-0259-x)
Supplement: Supplementary file 7 — Supplementary Table S1 [file 41396_2018_259_MOESM7_ESM.docx]

Table S1. Description of trap samples collected and analyzed in this study. *Trap was closed at 23:00 local time, 5 h 51 min after deployment.

| Trap number | Trap recovery date | Trap recovery location (Lat, Long) | Trap recovery time (local time) | Trap deployment duration | Number of individual particles analyzed |
| --- | --- | --- | --- | --- | --- |
| D1 | 2015_07_28 | 24° 36.5 N, 156° 37.7 W | 06:30 | 6 h 32 min | 9 |
| D2 | 2015_07_28 | 24° 37.9 N, 156° 35.5 W | 11:35 | 5 h 38 min | 11 |
| D3 | 2015_07_28 | 24° 39.2 N, 156° 33.0 W | 17:09 | 5 h 3 min | 12 |
| D4 | 2015_07_29 | 24° 39.1 N, 156° 28.6 W | 06:20* | 12 h 47 min | 11 |
| D5 | 2015_07_29 | 24° 40.0 N, 156° 26.9 W | 11:24 | 4 h 47 min | 12 |
| D6 | 2015_07_29 | 24° 40.3 N, 156° 24.5 W | 16:38 | 4 h 55 min | 12 |
| D7 | 2015_07_29 | 24° 40.2 N, 156° 22.2 W | 23:30 | 6 h 27 min | 9 |
