# Supplementary material for: Diverse diazotrophs are present on sinking particles in the North Pacific Subtropical Gyre
Source: ISME J. 2018 Aug 16;13(1):170–82. doi: 10.1038/s41396-018-0259-x (PMC6299005; doi:10.1038/s41396-018-0259-x)
Supplement: Supplementary file 8 — Supplementary Table S2 [file 41396_2018_259_MOESM8_ESM.docx]

Table S2. List of samples with numbers of high quality sequence reads for 16S rRNA and *nifH* gene libraries. na; No amplification

| SampleID | Sample type | Depth of collection (m) | Net trap number or seawater sample; size fraction | Number of 16S rRNA gene sequences | Number of *nifH* gene sequences |
| --- | --- | --- | --- | --- | --- |
| 63289 | Bulk particle | 150 | D1 | 34 963 | 72 526 |
| 63291 | Bulk particle | 150 | D2 | 37 083 | 89 377 |
| 63293 | Bulk particle | 150 | D3 | 33 910 | 63 257 |
| 63295 | Bulk particle | 150 | D4 | 39 167 | 101 542 |
| 63304 | Bulk particle | 150 | D5 | 33 616 | 76 694 |
| 63305 | Bulk particle | 150 | D6 | 39 267 | 89 655 |
| 63307 | Bulk particle | 150 | D7 | 37 299 | 101 329 |
| N3 | Individual particle | 150 | D1 | 23 750 | na |
| N4 | Individual particle | 150 | D1 | 23 116 | na |
| N5 | Individual particle | 150 | D1 | 31 524 | na |
| N6 | Individual particle | 150 | D1 | 15 058 | na |
| N7 | Individual particle | 150 | D1 | 18 909 | na |
| N8 | Individual particle | 150 | D1 | 25 774 | 61 925 |
| N9 | Individual particle | 150 | D1 | 13 340 | 56 173 |
| N10 | Individual particle | 150 | D1 | 14 877 | 48 539 |
| N11 | Individual particle | 150 | D1 | 16 204 | na |
| N23 | Individual particle | 150 | D2 | 17 634 | na |
| N24 | Individual particle | 150 | D2 | 16 725 | na |
| N25 | Individual particle | 150 | D2 | 9 815 | na |
| N26 | Individual particle | 150 | D2 | 22 883 | na |
| N27 | Individual particle | 150 | D2 | 16 788 | 52 865 |
| N29 | Individual particle | 150 | D2 | 25 867 | na |
| N30 | Individual particle | 150 | D2 | 19 402 | 55 417 |
| N31 | Individual particle | 150 | D2 | 12 672 | 79 098 |
| N32 | Individual particle | 150 | D2 | 21 251 | na |
| N33 | Individual particle | 150 | D2 | 20 547 | na |
| N34 | Individual particle | 150 | D2 | 24 248 | 60 393 |
| N37 | Individual particle | 150 | D3 | 28 957 | 52 304 |
| N38 | Individual particle | 150 | D3 | 24 331 | 61 053 |
| N39 | Individual particle | 150 | D3 | 18 379 | 49 699 |
| N40 | Individual particle | 150 | D3 | 15 453 | 49 334 |
| N41 | Individual particle | 150 | D3 | 22 167 | 57 285 |
| N42 | Individual particle | 150 | D3 | 24 136 | 56 117 |
| N43 | Individual particle | 150 | D3 | 37 557 | 58 644 |
| N44 | Individual particle | 150 | D3 | 26 535 | 66 701 |
| N45 | Individual particle | 150 | D3 | 19 471 | 76 495 |
| N46 | Individual particle | 150 | D3 | 24 650 | 53 692 |
| N47 | Individual particle | 150 | D3 | 15 667 | 66 266 |
| N48 | Individual particle | 150 | D3 | 16 241 | 60 534 |
| N60 | Individual particle | 150 | D4 | 21 266 | na |
| N61 | Individual particle | 150 | D4 | 22 702 | na |
| N62 | Individual particle | 150 | D4 | 27 743 | 48 173 |
| N63 | Individual particle | 150 | D4 | 17 265 | na |
| N64 | Individual particle | 150 | D4 | 29 091 | 58 858 |
| N65 | Individual particle | 150 | D4 | 14 240 | na |
| N66 | Individual particle | 150 | D4 | 13 455 | na |
| N67 | Individual particle | 150 | D4 | 7 583 | na |
| N68 | Individual particle | 150 | D4 | 14 118 | na |
| N69 | Individual particle | 150 | D4 | 11 488 | na |
| N70 | Individual particle | 150 | D4 | 13 814 | na |
| N92 | Individual particle | 150 | D5 | 23 959 | na |
| N93 | Individual particle | 150 | D5 | 31 517 | 56 956 |
| N94 | Individual particle | 150 | D5 | 22 412 | 47 691 |
| N95 | Individual particle | 150 | D5 | 17 542 | na |
| N96 | Individual particle | 150 | D5 | 12 365 | 74 984 |
| N97 | Individual particle | 150 | D5 | 15 423 | na |
| N98 | Individual particle | 150 | D5 | 19 731 | 70 288 |
| N99 | Individual particle | 150 | D5 | 24 451 | 69 389 |
| N100 | Individual particle | 150 | D5 | 14 060 | 71 014 |
| N101 | Individual particle | 150 | D5 | 15 778 | 47 716 |
| N102 | Individual particle | 150 | D5 | 19 547 | 80 182 |
| N103 | Individual particle | 150 | D5 | 19 617 | 47 164 |
| N124 | Individual particle | 150 | D6 | 42 905 | 81 473 |
| N125 | Individual particle | 150 | D6 | 24 407 | 58 410 |
| N126 | Individual particle | 150 | D6 | 12 566 | 57 234 |
| N127 | Individual particle | 150 | D6 | 14 655 | 68 396 |
| N128 | Individual particle | 150 | D6 | 20 951 | 76 320 |
| N129 | Individual particle | 150 | D6 | 24 595 | 72 999 |
| N130 | Individual particle | 150 | D6 | 21 653 | 53 428 |
| N131 | Individual particle | 150 | D6 | 19 407 | 45 718 |
| N132 | Individual particle | 150 | D6 | 14 052 | 37 293 |
| N133 | Individual particle | 150 | D6 | 25 894 | 73 530 |
| N134 | Individual particle | 150 | D6 | 14 171 | 60 813 |
| N135 | Individual particle | 150 | D6 | 16 569 | 72 950 |
| N156 | Individual particle | 150 | D7 | 27 098 | na |
| N157 | Individual particle | 150 | D7 | 23 124 | na |
| N158 | Individual particle | 150 | D7 | 20 296 | 56 930 |
| N159 | Individual particle | 150 | D7 | 23 163 | 51 118 |
| N161 | Individual particle | 150 | D7 | 27 896 | 62 553 |
| N162 | Individual particle | 150 | D7 | 17 000 | 78 628 |
| N163 | Individual particle | 150 | D7 | 29 868 | 87 501 |
| N166 | Individual particle | 150 | D7 | 26 218 | 55 492 |
| N167 | Individual particle | 150 | D7 | 24 953 | 52 556 |
| 63321 | Seawater | 150 | S1; >10 µm | 21 584 | 62 059 |
| 63322 | Seawater | 150 | S1; 0.2 - 10 µm | 28 194 | 46 754 |
| 63323 | Seawater | 150 | S1; >3 µm | 21 053 | 72 091 |
| 63324 | Seawater | 150 | S1; 0.2 - 3 µm | 29 105 | 20 957 |
| 63352 | Seawater | 150 | S2; >10 µm | 21 605 | 62 893 |
| 63353 | Seawater | 150 | S2; 0.2 - 10 µm | 97 892 | 44 423 |
| 63354 | Seawater | 150 | S2; >3 µm | 36 384 | 62 877 |
| 63355 | Seawater | 150 | S2; 0.2 - 3 µm | 105 840 | 27 326 |
| Zehr257 | Seawater | 5 | >0.2 µm | na | 14393 |
| Zehr258 | Seawater | 15 | >0.2 µm | na | 19703 |
| Zehr259 | Seawater | 25 | >0.2 µm | na | 20333 |
| Zehr260 | Seawater | 35 | >0.2 µm | na | 28227 |
| Zehr261 | Seawater | 45 | >0.2 µm | na | 23668 |
| Zehr262 | Seawater | 60 | >0.2 µm | na | 30954 |
| Zehr263 | Seawater | 75 | >0.2 µm | na | 32403 |
| Zehr264 | Seawater | 100 | >0.2 µm | na | 31150 |
| **Total** |  |  |  | **2 181 498** | **4 192 882** |
